# Pseudovirus Nanoparticles Displaying the Receptor-Binding Glycoprotein of Marburg Virus as Potential Vaccine Candidates

**DOI:** 10.3390/pathogens15101087

**Published:** 2026-10-16

**Authors:** Ming Xia, Frank S. Vago, Ming Tan

**Affiliations:** 1 Division of Infectious Diseases, Cincinnati Children’s Hospital Medical Center, Cincinnati, OH 45229, USA; ming.xia@cchmc.org; 2 Department of Biological Sciences, Purdue University, West Lafayette, IN 47907, USA; fvago@purdue.edu; 3 Department of Pediatrics, University of Cincinnati College of Medicine, Cincinnati, OH 45229, USA

**Keywords:** Marburg virus, Marburg virus vaccine, norovirus S nanoparticle, pseudovirus nanoparticle, receptor-binding glycoprotein

## Abstract

Background: The lack of a licensed vaccine against the deadly Marburg virus (MARV) calls for the development of a novel and effective vaccine strategy. Methods: We report here our development of three pseudovirus nanoparticles (PVNPs), each displaying a MARV-neutralizing glycoprotein (GP) segment of different lengths, as MARV vaccine candidates. These PVNPs, designated S-GP_278_, S-GP_377_, and S-GP_486_, were generated using our proprietary norovirus shell nanoparticle (SNP) platform. The PVNPs were evaluated for their production, self-assembly, morphology, structural properties, and immunogenicity. Results: Our data demonstrated that the three recombinant S-GP proteins, S-GP_278_, S-GP_377_, and S-GP_486_, can be produced at low cost using an *E. coli* expression system. The purified recombinant S-GP proteins self-assembled into PVNPs with high efficacy. Structural modeling of the S-GP1 PVNPs highlighted their key architectural features, consisting of an icosahedral inner shell and multiple trimeric GP structures displayed on the surface. Immunization of mice with the S-GP PVNPs elicited high titers of GP-specific antibodies, including IgG and IgA. Conclusions: Collectively, these data support the notion that the S-GP PVNPs may serve as potential MARV vaccine candidates worthy of further development.

## 1. Introduction

Marburg viruses (MARVs), like Ebolaviruses (EBOVs), cause hemorrhagic fever characterized by symptoms such as fever, severe headache, muscle pain, vomiting, diarrhea, and internal or external bleeding. Although both MARVs and EBOVs belong to the *Filoviridae* family, they are classified into two distinct genera: *Marburgvirus* and *Ebolavirus* [1]. Marburg virus disease (MVD), also known as Marburg hemorrhagic fever, is a severe and often fatal illness in humans, with an overall case fatality rate of approximately 50%, ranging from 24% to 88% in past outbreaks [1,2]. The Egyptian fruit bat (*Rousettus aegyptiacus)*, a member of the *Pteropodidae* family, is believed to be the primary nature reservoir of MARVs. Transmission to humans occurs through zoonotic spillover events, and subsequent human-to-human transmission can lead to deadly MVD outbreaks. The most recent MVD outbreak occurred in October 2024 in Rwanda, where at least 64 individuals were infected, resulting in 15 deaths [3]. Currently there are no approved vaccines or antiviral treatments for MVD. Supportive care, including rehydration and symptomatic management, can improve chances of survival [2].

MARVs are enveloped RNA viruses with a complex structure. They are pleomorphic, exhibiting various shapes such as filamentous, rod- or ring-like, crook- or 6-shaped, or branched forms, with a diameter of approximately 80 nm and of variable lengths [4]. The viral core is the helical nucleocapsid, a tubular structure composed of the viral RNA genome and several associated proteins. This nucleocapsid features boomerang-shaped protrusions that spiral along its outer surface [5]. The single-stranded, negative-sense RNA genome, approximately 19.1 kilobases in length, contains seven genes encoding seven viral proteins in the following order from the 3′- to 5′-end: nucleoprotein (NP), viral protein 35 (VP35), VP40, glycoprotein (GP), VP30, VP24, and RNA-dependent RNA polymerase (L). Among these, VP40 and VP24 form the viral matrix that encases the nucleocapsid [1,6]. The surface of the virus is adorned with spikes that bind to receptors on host cells, facilitating viral attachment and fusion with the host cell membrane, thereby enabling viral entry [1]. As a result, these viral spikes serve as an ideal target for vaccination.

The MARV spike is a trimeric protein complex composed of three acylated and highly glycosylated GP molecules. Classified as a type I transmembrane protein, GP is divided into two major subunits, GP1 and GP2, which are generated by the furin-mediated cleavage of a precursor protein within the Golgi network of host cells [7]. GP1 contains a heavily glycosylated mucin-like domain that interacts with host receptors, whereas GP2 includes a membrane anchor and a fusion loop that facilitate membrane fusion [8]. Accordingly, GP1 is referred to as the receptor-binding domain, and GP2 as the fusion domain. Because of its critical role in initiating viral infection, GP is an excellent target for vaccine development, aiming to elicit neutralizing antibodies that inhibit the interaction between the GP1 and host receptors and block the fusion activity of GP2, thereby preventing MARV entry and infection.

Although no commercial vaccines for MARV are currently available, multiple vaccine candidates are under development. The World Health Organization (WHO) has prioritized four viral vector-based vaccine candidates targeting the GPs for evaluation in human trials, based on their safety data, immune responses, and protective efficacy in animal models [9]. The most advanced candidate, currently in a phase II clinical trial in Uganda and Kenya [10] (ClinicalTrials.gov ID: NCT05817422), is a monovalent chimpanzee adenoviral-vectored MARV vaccine developed by the Sabin Vaccine Institute. This follows a phase I trial demonstrating its safety, as well as its ability to elicit rapid and robust immune responses [11]. The second prioritized MARV vaccine candidate, known as the ChAdOx1 Marburg vaccine, is also an adenoviral vector-based vaccine developed at Oxford University. A phase 1 trial of this candidate was performed in early 2024 [12]. The remaining two prioritized MARV vaccine candidates are vesicular stomatitis virus (VSV) vector-based vaccines. The first, named rVSV∆G-MARV-GP, was developed by the International AIDS Vaccine Initiative (IAVI) [13], while the second, named rVSV∆G-MARV (Angola) GP, was developed by Public Health Vaccines LLC [14]. Both vaccine candidates are currently in preclinical development.

Our prior studies demonstrated that the modified norovirus shell (S) domain self-assembles into S nanoparticles (SNPs), which resemble the inner shell of the norovirus and feature flexible C-terminal hinges on the surface [15,16]. Importantly, fusing neutralizing antigens from other pathogens to the hinges of the S domain results in the self-assembly of pseudovirus nanoparticles (PVNPs), with the antigens displayed on the surface [15,16,17,18,19,20]. This unique PVNP architecture and antigen presentation markedly enhances the immunogenicity of the displayed antigens. Building on this SNP platform, we employed a novel MARV vaccine development strategy in this study by generating a new type of PVNP targeting MARV spikes. This was achieved by fusing the MARV GP, in varying lengths, to the norovirus shell (S) domain and producing the fusion proteins using a recombinant protein expression system. The resulting S-GP fusion proteins self-assembled into PVNPs and elicited robust GP-specific IgG and IgA antibody responses. Therefore, these S-GP PVNPs show strong potential as MARV vaccine candidates.

## 2. Materials and Methods

### 2.1. Plasmid Constructs for the Expressions of the S-GP Fusion Proteins

Three protein sequences of the GP antigen from the MARV Angola strain (GenBank accession #s: AIL25245.1 and KM261523.1) were selected for this study. The first sequence (GP_278_) contains 278 amino acids of GP1, spanning residues L19 to P296. The second sequence (GP377) comprises 377 residues, including 172 amino acids of GP1 (L19 to N190) and 205 residues of GP2 (R432 to T636). The third fragment (GP486) is 486 amino acids in length, containing 278 amino acids of GP1 (from L19 to P296) and 208 residues of GP2 (V429 to T636).

**Figure 1:**
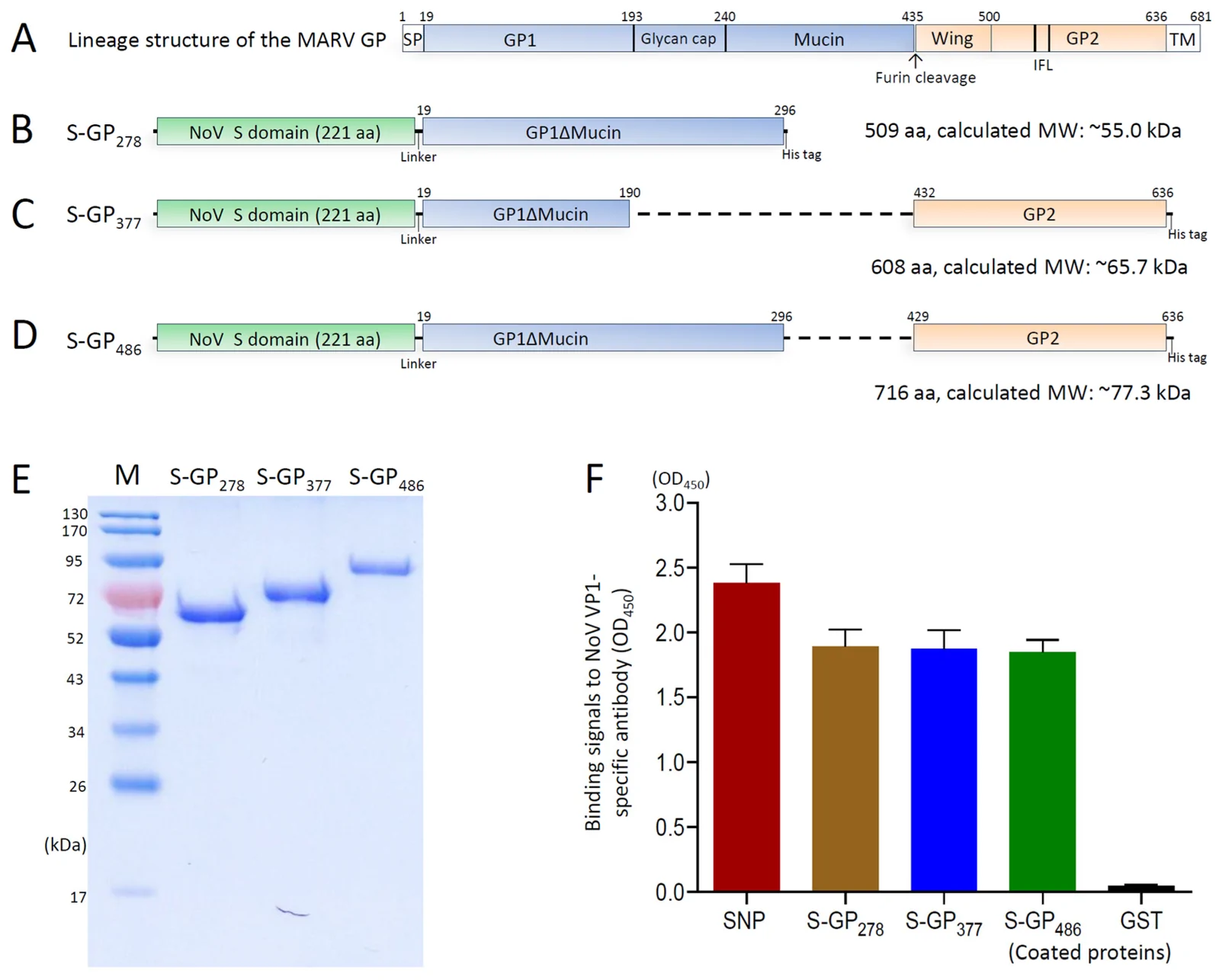
Lineage structure of the Marburg virus (MARV) glycoprotein (GP), constructs of the three S-GP fusion proteins, and production of these S-GP proteins. (**A**) Schematic illustration of the MARV GP lineage structure. SP, signal peptide; mucin, mucin-like domain; IFL, internal fusion loop; TM, transmembrane domain. Numbers above the illustrations indicate amino acid positions. (**B**–**D**) Schematic illustration of the three S-GP fusion proteins (S-GP_278_, S-GP_377_, and S-GP_486_) generated in this study, shown in comparison with MARV GP. The total lengths and calculated molecular weights (MWs) of the three S-GP fusion proteins are indicated beside panel B or below panels C and D. (**E**) SDS-PAGE analysis of the three purified S-GP fusion proteins. M, prestained protein markers with indicated MWs (kDa). (**F**) EIA analysis showing that the three S-GP fusion proteins strongly bound the guinea pig hyperimmune serum raised against norovirus (NoV) VLPs containing the S domain. The S-domain protein (S) served as the positive control, whereas glutathione S-transferase (GST) served as the negative control.

The DNA sequences corresponding to these three GP antigens were codon-optimized to *Escherichia coli* and synthesized by GenScript (Piscataway, NJ, USA). Each synthesized DNA fragment was cloned into a previously constructed pET-24b (Novagen, WI, USA)-based plasmid designed for expressing the S-HA1 fusion protein of an H7N9 influenza virus [20], replacing the HA1-encoding region. A GGGG linker was inserted between the S domain with the hinge and the GP antigen, and a His-tag was added at the C-terminus of the GP antigen for protein purification (Figure 1A).

### 2.2. S-GP Fusion Protein Expression and Purification

The three His-tagged recombinant S-GP fusion proteins were expressed in *E. coli* BL21 (DE3) cells. The target proteins were isolated from the bacterial lysates under denaturing conditions as described earlier [17]. After induction with 0.4 mM isopropyl ß-D-1-thiogalactoyranoside (IPTG) for approximately 16 h, the harvested bacteria were resuspended with 6 M guanidine hydrochloride (GnHCl) in phosphate buffer–NaCl (50 mM NaPO_4_, 300 mM NaCl, pH 7.2). Following bacteria lysis by sonication, the target proteins in the clarified bacterial lysates were purified using cobalt resins via their His-tags. To remove the GnHCl, the purified proteins were sequentially dialyzed for 16 h each against phosphate buffer (50 mM NaPO_4_, 300 mM NaCl, pH 7.4) containing 6M, 4M, 2M and 1M urea, respectively. For protein refolding, the proteins in phosphate buffer with 1M urea were dialyzed overnight against redox buffer (100 mM Tris, 400 mM L-arginine, 2 mM EDTA, 5 mM reduced glutathione, 1 mM oxidized glutathione, pH 8.0).

### 2.3. Sodium Dodecyl Sulfate Polyacrylamide Gel Electrophoresis (SDS-PAGE)

The purified recombinant S-GP proteins were analyzed by SDS-PAGE using 12% resolving gels, following the method outlined in a previous study [21].

### 2.4. Gel-Filtration Chromatography

Gel-filtration chromatography was conducted using an ÄKTA Fast-Performance Liquid Chromatography system (FPLC, ÄKTA pure^TM^ 25 L, GE Healthcare Life Sciences, Uppsala, Sweden) equipped with a size-exclusion column (Superdex 200, 10/300 GL, 25 mL bed volume, GE Healthcare Life Sciences). The column was calibrated using gel-filtration calibration kits (GE Healthcare Life Sciences). The elution peaks of the S-GP PVNPs in the void volume, along with their potential monomeric or oligomeric forms, were evaluated by comparison with previously prepared S-HA1 H7 PVNPs (~3.12 MDa) [20] and glutathione S-transferase (GST)-dimers (54 kDa) [22], respectively. Relative protein concentrations in the eluate were determined by measuring the absorbance at 280 nm (A_280_).

### 2.5. Transmission Electron Microscopy (TEM)

Negative-stain TEM was employed to examine the morphology of the S-GP PVNPs as previously described [16]. The purified S-GP proteins were applied to TEM grids (FCF200-CV-50, Electron Microscopy Sciences, Hatfield, PA, USA) and stained with 1% ammonium molybdate. The stained grids were air-dried and imaged using a Hitachi microscope (model H-7650, Hitachi High-Tech Corporation, Tokyo, Japan) at 80 kV, with magnifications ranging from 15,000× to 40,000×.

### 2.6. Dynamic Light Scattering (DLS)

Fifty microliters of each S-GP protein sample were loaded to a specific cuvette (MOS Plastics Inc., San Jose, CA, USA) and analyzed using the DynaPro nanostar DLS instrument (Wyatt Technology, Santa Barbara, CA, USA). Size distributions of the S-GP PVNPs were calculated using DYNAMICS software (version 7.10, Wyatt Technology).

### 2.7. Structural Modeling of the S-GP PVNPs

Structural models of the S-GP PVNPs with T = 1, T = 3, and T = 4 icosahedral symmetries were generated using UCSF ChimeraX software (version 1.7) [23], following the approach described previously [18]. The electron density map from cryogenic electron microscopy (cryoEM) of the S-HA1 H7 PVNP [20] served as the template. Known structures of norovirus inner shells with T = 1, T = 3, or T = 4 icosahedral symmetries [17,24,25,26], along with MARV GP protein trimers (PDB code: 5UQY), were docked into the corresponding regions of the template. Structural assessment and image rendering of the S-GP PVNP models were also carried out in UCSF ChimeraX (version 1.12).

### 2.8. Enzyme Immunoassays (EIAs)

The identity of the purified S-GP proteins was determined by EIA assays. Briefly, the three recombinant S-GP proteins were coated onto 96-well microtiter plates at a concentration of ~2 ng/µL, with the S-domain protein (S nanoparticle, SNP) used as a positive control and glutathione S-transferase (GST) as a negative control. Following blocking with 5% nonfat milk, the coated proteins were incubated with guinea pig hyperimmune serum generated by immunization with norovirus VLPs containing the S domain [27] at a 1:3000 dilution. Bound antibodies were detected using a goat anti-guinea pig IgG-Horseradish Peroxidase (HRP) conjugate (1:4000, MP Biomedicals, Irvine, CA, USA). The HRP signal was developed by adding substrates, and binding signals of the S-GP protein to the S-domain-specific antibodies were quantified at an optical density (OD) of 450 nm.

### 2.9. Mouse Immunization

BALB/c mice purchased from the Jackson Laboratory (Bar Harbor, ME, USA) were housed under pathogen-free conditions in the Division of Veterinary Services at Cincinnati Children’s Hospital Medical Center (CCHMC). A total of 40 female mice, approximately 8 weeks old, were randomly assigned to four groups (n = 8 per group). Each group received one of the following immunogens: (1) S-GP_278_ PVNPs; (2) S-GP_377_ PVNPs; (3) S-GP_486_ PVNPs; (4) the SNP platform control; (5) the phosphate-buffered saline (PBS) control. Each immunogen was administered at a dose of 10 μg/mouse, mixed with Imject Alum Adjuvant (aluminum hydroxide, Thermo Fisher Scientific, Waltham, MA, USA, 40 mg/mL) at a 1:1 volume ratio, as reported in a prior study [28]. The formulated immunogen was delivered intramuscularly into the thigh muscle at a volume of approximately 80 µL/mouse/dose. Immunizations were conducted three times at two-week intervals. Blood samples were collected from the tail vein two weeks after the second immunization and via cardiac puncture two weeks after the final immunization [21], respectively, for serum preparation.

### 2.10. Determination of GP-Specific Antibody Titers

GP-specific IgG and IgA antibody titers in mouse sera following immunization with the S-GP PVNPs and controls were measured using enzyme-linked immunosorbent assays (ELISAs), as described previously [20]. Commercial recombinant GP protein lacking the transmembrane domain of the MARV Angola strain (IBT Bioservices, Rockville, MD, USA) was coated onto 96-well microtiter plates at a concentration of 0.25 ng/µL as the capture antigen. This GP protein was produced in Sf9 insect cells using a baculovirus expression system and purified by FPLC. After blocking with 5% nonfat milk, serial two-fold dilutions of mouse sera were added. Bound IgG was measured using the goat anti-mouse IgG-HRP conjugate (1:5000, MP Biomedicals), and bound IgA was detected using the goat anti-mouse IgA-HRP conjugate (1:1000, Bio-Rad, Hercules, CA, USA). GP-specific IgG and IgA titers were defined as the highest serum dilutions yielding positive signals (OD_450_ ≥ 0.2). For comparisons, S-GP PVNP-specific and SNP-specific IgG and IgA antibody titers were also evaluated using the S-GP_486_ PVNPs and SNPs as capture antigens, respectively, following the same procedure described above.

### 2.11. Ethics Statement

All animal experiments were conducted in accordance with the recommendations outlined in the Guide for the Care and Use of Laboratory Animals (23a) by the National Institute of Health (NIH). All procedures were reviewed and approved by the Institutional Animal Care and Use Committee (IACUC) of the Cincinnati Children’s Hospital Research Foundation (Animal Welfare Assurance No. A3108-01).

### 2.12. Statistical Analysis

Statistical comparisons between two datasets were carried out in GraphPad Prism 10.0 (GraphPad Software, Inc. San Diego, CA, USA) using an unpaired *t*-test. Statistical significance was defined as follows: non-significant (ns), *p* > 0.05; significant (*), *p* < 0.05; highly significant (**), *p* < 0.01; very significant (***), *p* < 0.001; extremely significant (****), *p* < 0.0001.

## 3. Results

### 3.1. Generation of Three S-GP Fusion Proteins

Three norovirus (NoV) shell (S) domain-based fusion proteins containing MARV GP sequences of different lengths were generated using an *E. coli* expression system. The proteins were designated as: (1) S-GP_278_, consisting of the NoV S domain and GP1 lacking most of the mucin-like domain; (2) S-GP_377_, consisting of the NoV S domain, the first half of GP1, and GP2; (3) S-GP_486_, consisting of the NoV S domain, GP1 lacking the most of the mucin-like domain, and GP2 (Figure 1A–D). The fusion proteins were purified using His-tag binding cobalt resin under denaturing conditions. Denaturing reagents were subsequently removed from the proteins by serial dialysis. After refolding in a redox refolding buffer, soluble proteins free of denaturants were obtained, with yields ranging from 20 to 30 mg of target protein per liter of bacterial culture.

SDS-PAGE analysis of the refolded proteins showed a single major band for each S-GP fusion protein (Figure 1E), indicating high purity (>90%) of the purified proteins. Notably, these proteins exhibited slower electrophoretic mobilities, resulting in apparent molecular weights (MWs) larger than their calculated values (Figure 1, compare panels B to D with E). Specifically, the three proteins migrated to approximately 60, 75, and 85 kDa on the SDS-PAGE gel, which are larger than their predicted MWs of approximately 55, 66, and 77 kDa, respectively. The S-domain identity of the S-GP proteins was confirmed by EIA using guinea pig hyperimmune serum generated by immunization with norovirus VLPs containing the S domain [27] as the detection antibody. All three S-GP proteins, as well as the S-domain protein used as a positive control, were strongly recognized by the hyperimmune serum, whereas GST, which lacks the S domain, showed no detectable binding signals (Figure 1F).

### 3.2. Self-Assembly of the S-GP Proteins into PVNPs

**Figure 2:**
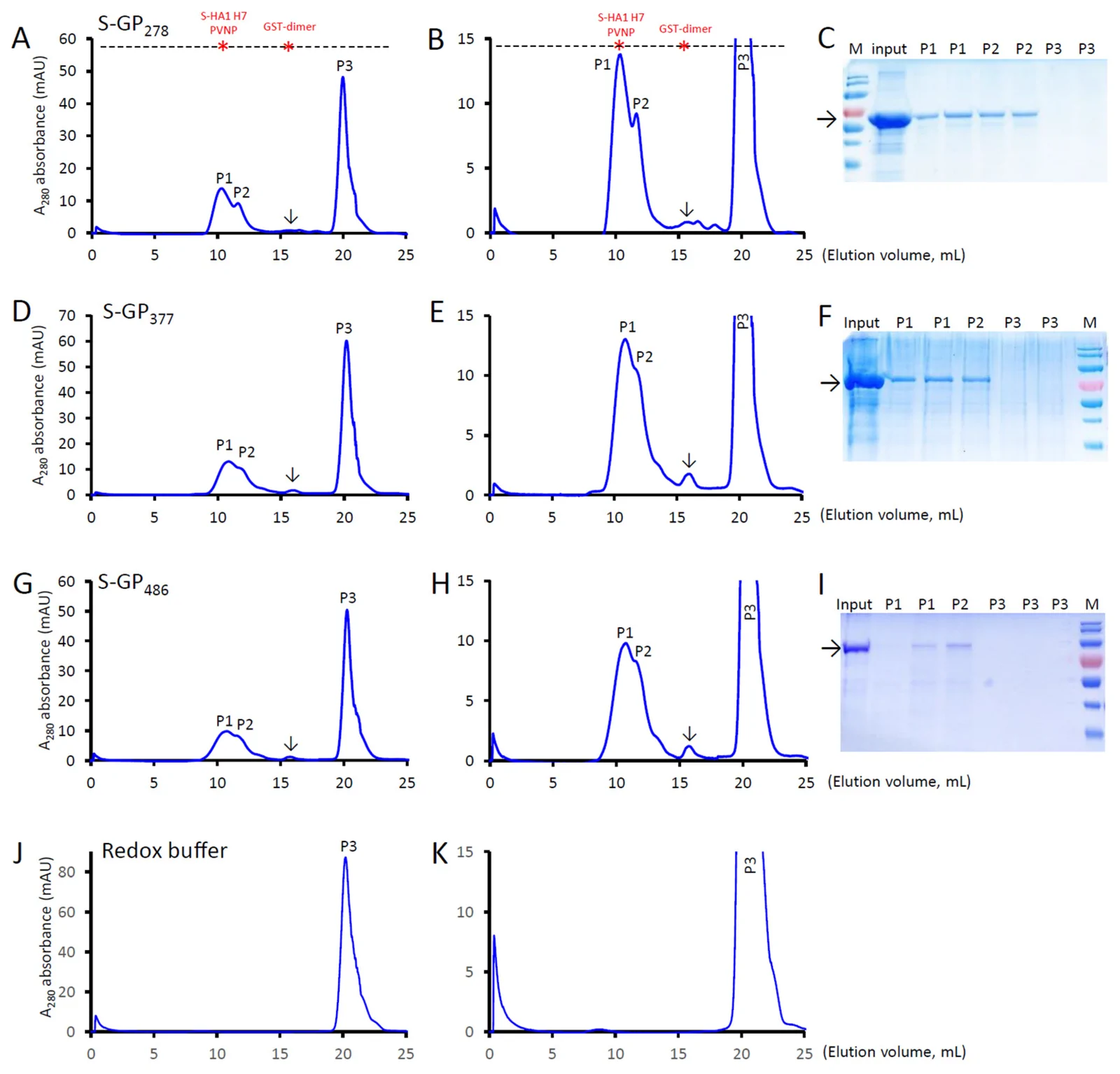
Analysis of the three S-GP fusion proteins by gel-filtration chromatography and SDS-PAGE. Representative elution profiles from gel-filtration chromatography of S-GP_278_ (**A**,**B**), S-GP_377_ (**D**,**E**), and S-GP_486_ (**G**,**H**) proteins. Panels (**B**,**E**,**H**) are enlargements of panels (**A**,**D**,**G**), respectively, focusing on the first two overlapping peaks (P1 and P2) in and near the void volume. The elution peaks corresponding to the S-GP PVNPs (P1 and P2) were identified based on the elution position of S-HA1 H7 PVNPs, whereas the minor peaks representing monomeric or dimeric S-GP protein (indicated by arrows) were determined by the elution position of the GST dimer, indicated by red stars on the dashed reference lines above the elution curves. The Y-axes show A_280_ absorbance (mAU), and the X-axes show accumulative elution volume (mL). Relative protein abundance within each peak is reflected by A_280_ absorbance. The three major elution peaks (P1, P2, and P3) from gel-filtration chromatography were analyzed by SDS-PAGE (**C**,**F**,**I**). Input, S-GP protein sample before loading onto the gel-filtration column; M, prestained protein markers with indicated molecular weights (kDa). Representative elution profiles from gel-filtration chromatography of redox refolding buffer without S-GP proteins (**J**,**K**).

The three refolded S-GP fusion proteins were analyzed by gel-filtration chromatography using a Superdex 200 size-exclusion column, followed by SDS-PAGE analysis of the elution peaks (Figure 2). All three proteins were eluted as two partially overlapping peaks in or near the void volume (Figure 2A,B,D,E,G,H, P1 and P2), indicating that the three proteins self-assembled into large complexes or particles with high MWs. Because the previously characterized S-HA1 PVNPs eluted at similar locations [18,20], we hypothesized that the three S-GP fusion proteins formed comparable PVNPs. The presence of two partially overlapping peaks suggested that the S-GP proteins may assemble into PVNPs of at least two distinct sizes. In contrast, peaks corresponding to the monomeric or dimeric S-GP proteins were minor (Figure 2B,E,H, shown by arrows), indicating that the vast majority of the proteins self-assembled into PVNPs. A prominent peak appearing after an elution volume of ~20 mL was observed for each protein (Figure 2A,B,D,E,G,H), but SDS-PAGE revealed no protein bands (Figure 2C,F,I). On the other hand, the redox refolding buffer alone displayed the same peak (Figure 2J,K), indicating that this peak originated from the buffer, most likely from component oxidized glutathione, which exhibits absorbance at 280 nm.

### 3.3. Morphologies of the S-GP PVNPs

**Figure 3:**
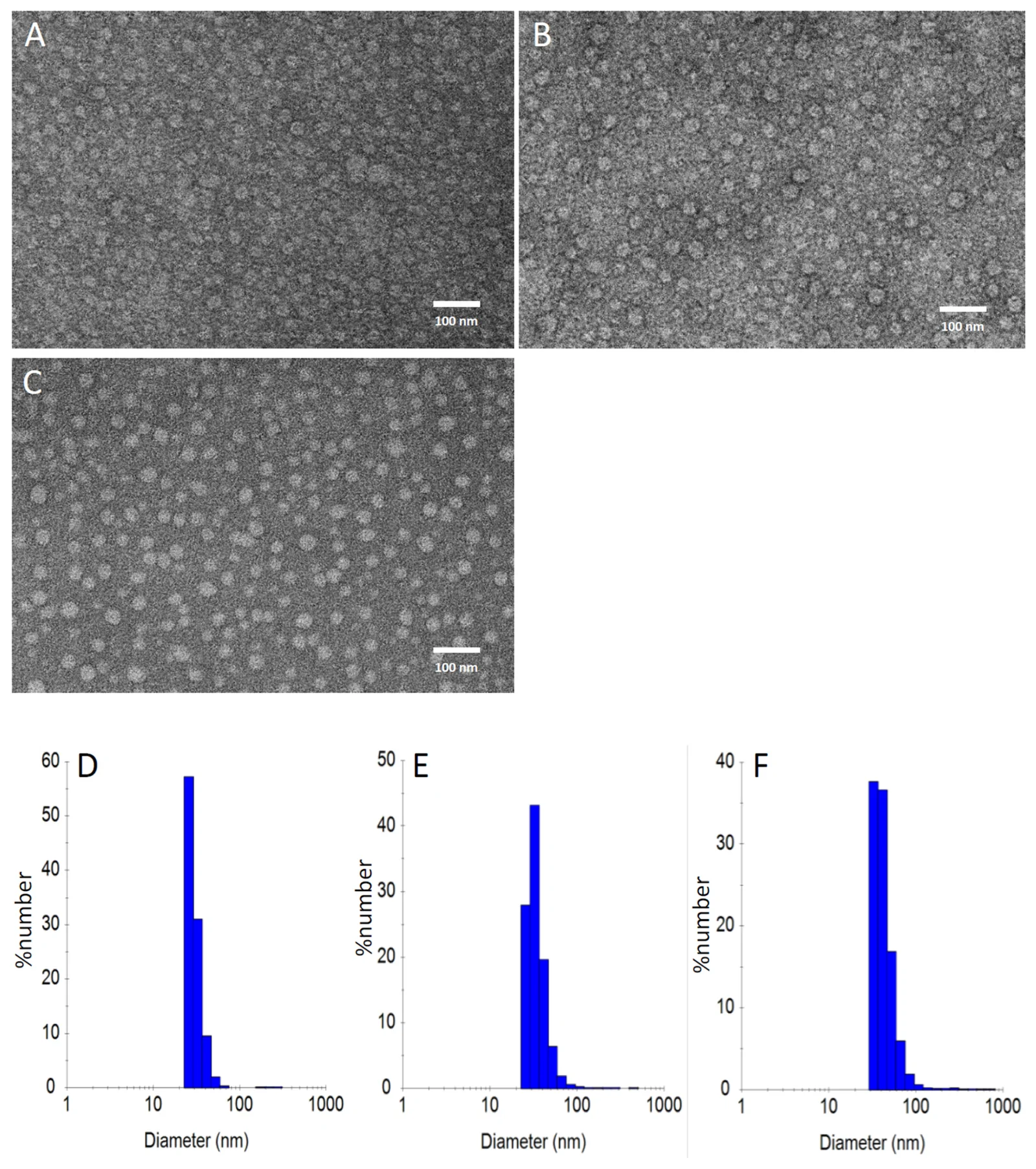
Morphologies and size distributions of the S-GP PVNPs. (**A**–**C**) Representative TEM micrographs of the three S-GP proteins, showing PVNPs assembled by S-GP_278_ (**A**), S-GP_377_ (**B**), and S-GP_486_ (**C**). A 100 nm scale bar is shown in each micrograph. (**D**–**F**) Size distributions of PVNPs assembled by S-GP_278_ (**D**), S-GP_377_ (**E**), and S-GP_486_ (**F**), determined by dynamic light scattering (DLS). The Y-axes indicate the percentage of particles, and the X-axes indicate particle diameter (nm).

To confirm that the S-GP proteins self-assembled into PVNPs and to examine their morphologies, protein samples from the two overlapping elution peaks (P1 and P2) obtained by gel-filtration chromatography (Figure 2) were analyzed using transmission electronic microscopy (TEM). TEM revealed typical PVNP morphologies for all three S-GP proteins, with particles of various sizes (Figure 3A–C). The predominant particle populations range from 25 to 45 nm in diameter, and the S-GP_486_ PVNPs appeared to form the largest particles overall (Figure 3C), consistent with their greater protein size among the three constructs.

### 3.4. Size Distributions of the S-GP PVNPs

To further characterize the size distributions of the S-GP PVNPs, the S-GP proteins were analyzed by dynamic light scattering (DLS). The data indicated that all three proteins formed PVNPs of various sizes, with peak distributions falling within the range observed by TEM, and with particle sizes following the order S-GP_486_ > S-GP_377_ > S-GP_278_. Specifically, the majority of the S-GP_278_ PVNPs ranged from 23 nm to 38 nm; the majority of the S-GP_377_ PVNPs spanned from 24 nm to 42 nm, and the majority of the S-GP_486_ PVNPs spanned from 29 nm to 48 nm in diameter, consistent with their relative protein sizes. These size variations are likely due to the known propensity of recombinant norovirus VP1 to assemble into T = 1, T = 3, and T = 4 VLPs in vitro [24,25,26]. Comparison of these PVNPs with established norovirus VLPs of T = 1, T = 3, and T = 4 icosahedral symmetry suggests that the PVNPs formed by the three S-GP proteins encompass all three symmetry types, corresponding to assemblies of 60 (T = 1), 180 (T = 3), and 240 (T = 4) fusion protein units, respectively.

### 3.5. Structural Modeling of S-GP1 PVNPs

**Figure 4:**
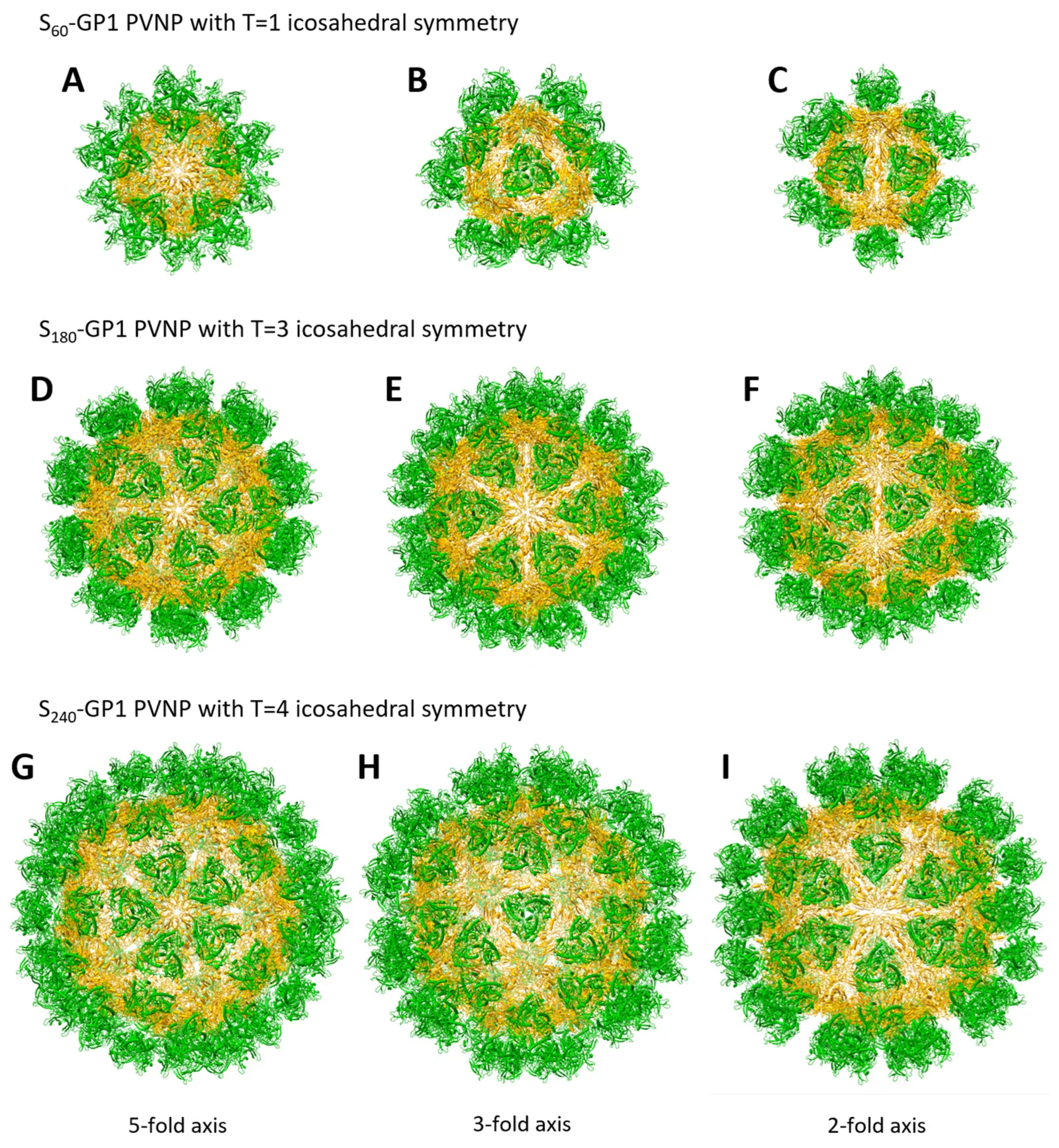
Structural models of S-GP1 PVNPs with different icosahedral symmetries. (**A**–**C**) Illustrations of the T = 1 S_60_-GP1 PVNP. (**D**–**F**) Illustrations of the T = 3 S_180_-GP1 PVNP. (**G**–**I**) Illustrations of the T = 4 S_240_-GP1 PVNP. The left panels (**A**,**D**,**G**) show views along the 5-fold axis; the middle panels (**B**,**E**,**H**) show views along the 3-fold axis; and the right panels (**C**,**F**,**I**) show views along the 2-fold axis. The interior shells of the S-GP1 PVNPs assembled by the S domains (S nanoparticle, SNP) are displayed in orange, while the trimeric GP1 protrusions are shown in green. All protein structures are rendered using cylinder/stud representations. These structures are computationally predicted models generated through in silico analyses and have not been experimentally determined or validated.

In a previous study, we solved the 3D structure of the S-HA1 PVNP of an avian H7N9 influenza virus by cryoEM [20]. It exhibits a T = 1 icosahedral symmetry and consists of 60 S-HA1 proteins, with 60 HA1 domains organized into 20 trimers on the particle surface. Because MARV GP proteins also form trimers and the crystal structure of the GP1 trimer has been elucidated [29] (PDB code: 5UQY), we generate S-GP1 structural models with T = 1, T = 3, and T = 4 icosahedral symmetry using the cryoEM structure of the S-HA1 PVNP as a template in UCSF ChimeraX software. As shown in Figure 4, the S-GP1 PVNP structures corresponding to T = 1, T = 3, and T = 4 symmetry represent particles of distinct sizes, consistent with those observed by TEM (Figure 3A–C) and DLS (Figure 3D–F). Specifically, the GP1 proteins form 20 (T = 1), 60 (T = 3), and 80 (T = 4) trimers protruding from the surface of their respective S-domain nanoparticles (Figure 4). Thus, the three structural models differ in icosahedral symmetries, particle sizes, numbers of S-GP fusion proteins, and the numbers of GP trimers on the surface. We assume that the three S-GP proteins assemble into similar PVNPs that share the same icosahedral symmetry and that all three GP proteins form comparable trimeric structures on the particle surface, given the strong propensity of the S domain to self-assemble into icosahedrons and the intrinsic trimeric nature of GP proteins.

### 3.6. Antibody Responses of the S-GP PVNPs

**Figure 5:**
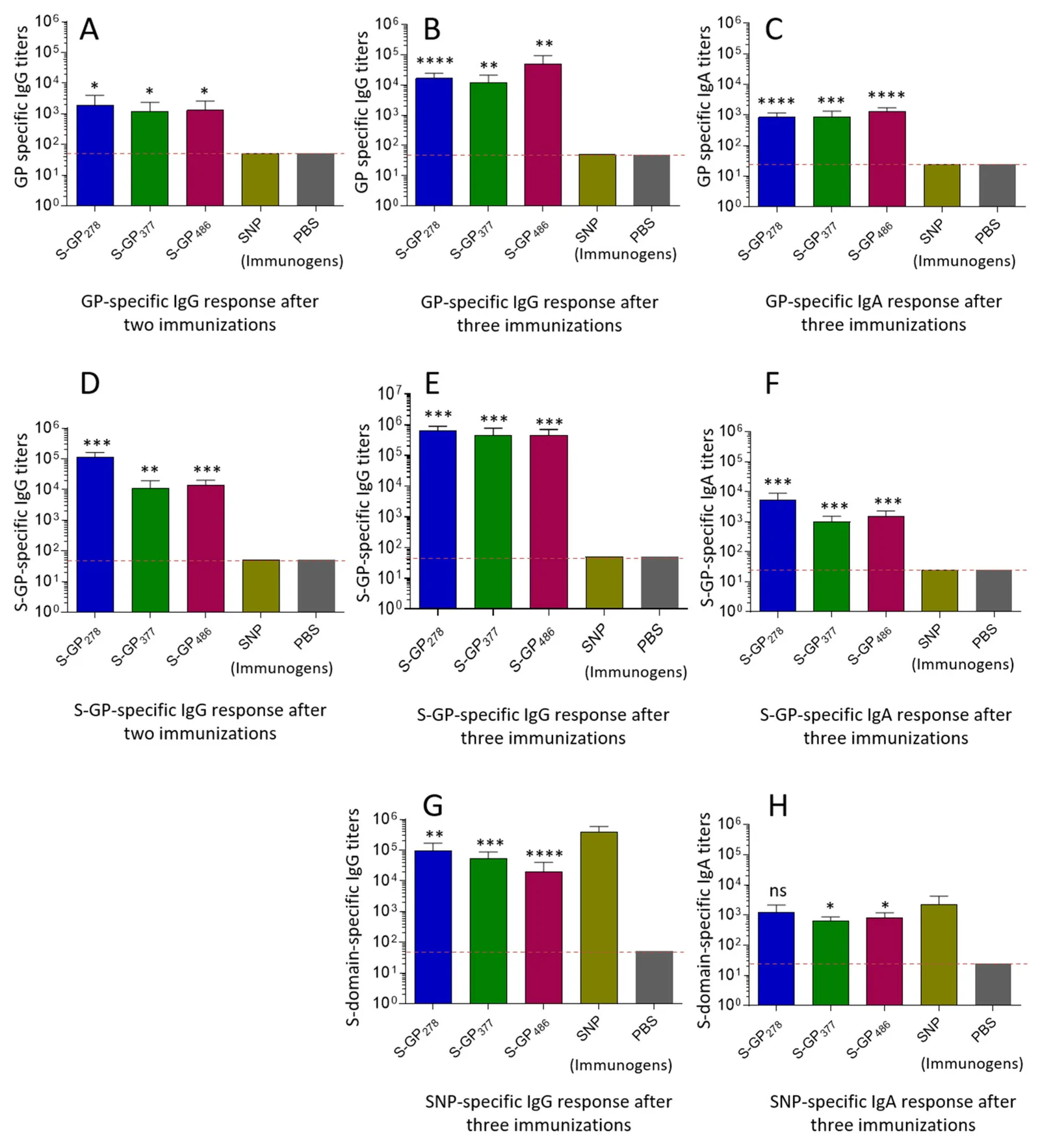
Serum IgG and IgA responses in mice following the administration of three S-GP PVNPs. (**A**–**C**) Glycoprotein (GP)-specific IgG (**A**,**B**) and IgA (**C**) titers after two (**A**) and three (**B**,**C**) administrations of the three S-GP PVNPs individually were evaluated by EIAs, using commercial GP protein as capture antigens. (**D**–**F**) S-GP-specific serum IgG (**D**,**E**) and IgA (**F**) titers after two (**D**) and three (**E**,**F**) immunization of the three S-GP PVNPs individually using recombinant S-GP_486_ PVNP as capturing antigens. (**G**,**H**) S-domain-specific IgG (**G**) and IgA (**H**) titers after three immunizations of the three S-GP PVNPs individually, using the recombinant shell nanoparticle (SNP) as capturing antigens. Mouse sera collected after administrations of the SNP or PBS alone served as controls. The Y-axes show antibody titers, all presented on a log 10 scale, while the X-axes indicate the corresponding immunogens. The dashed lines indicate the limit of detection (LOD). Statistical differences of the indicated columns relative to the SNP controls are denoted as “ns” for not significant (*p* > 0.05); “*” for significant (*p* < 0.05); “**” for highly significant (*p* < 0.01); and “***” or “****” for extremely significant (*p* < 0.001 or *p* < 0.0001).

Fourteen days following two or three intramuscular administrations of the individual S-GP PVNPs, using the S nanoparticle (SNP) as the platform control and PBS as negative control, GP-specific IgG and IgA antibody titers in mouse sera were evaluated by ELISA using commercial recombinant GP proteins (see the Materials and Methods Section) as the capture antigen. After two doses of immunization, GP-specific IgG titers increased significantly, reaching levels of 1:1200 to 1:2000 (Figure 5A). Following three immunizations, IgG titers rose by 9- to 30-fold, reaching levels of 1:16,800 to 1:38,400 (Figure 5B). In comparison, GP-specific serum IgA titers were lower, ranging from 1:850 to 1:1300 (Figure 5C). These data showed that the PVNP displaying the longest GP fragment (GP_486_) induced the strongest GP-specific IgG and IgA antibody responses, thus identifying it as the lead vaccine candidate for future development.

Using the recombinant S-GP_486_ PVNP as the capture antigen, we found that the measured IgG and IgA titers were substantially higher than those described above. After two administrations, IgG titers ranged from 1:10,800 to 1:110,933 (Figure 5D). Following three administrations, IgG titers increased to 1:345,600 to 1:742,400 (Figure 5E), representing a 6.7- to 50-fold increase compared with titers after two immunizations. IgA titers showed similar trends, ranging from 1:1200 to 1:6600, which were 1.4- to 7.7-fold higher than those measured using the commercial GP protein as the capture antigen (Figure 5F).

Finally, we assessed antibody responses generated by the SNP platform using previously prepared SNP [16] as the capture antigen. After three immunizations, SNP-specific IgG titers were moderate (1:20,000 to 1:94,400; Figure 5G), whereas IgA titers were lower (1:650 to 1:1250; Figure 5H), compared with the results described above. Antibody responses after two immunizations were evaluated only to a limited extent because only small volumes of blood could be collected from live mice via the tail vein.

## 4. Discussion

Leveraging our proprietary norovirus shell nanoparticle (SNP) platform, we developed three pseudovirus nanoparticles (PVNPs), each displaying a Marburg virus (MARV)-neutralizing glycoprotein (GP) fragment of a different length, as vaccine candidates against MARV. As part of the initial characterization of these PVNPs, we demonstrated that: (1) the three S-GP fusion proteins could be produced cost-effectively using a bacterial expression; (2) the purified S-GP fusion proteins efficiently self-assembled into their respective S-GP PVNPs; (3) the key architectural features of a typical PVNP were elucidated through structural modeling; (4) immunization with the PVNPs induced robust serum antibody responses in mice. Collectively, these findings support the potential of the S-GP PVNPs as vaccine candidates against MARV and warrant their further development and evaluation.

Among the three GP fragments displayed on the PVNPs, the 486-amino-acid GP fragment (GP_486_) represents the largest antigen ever presented on our SNP platform. When the N-terminal linker (GGGG) connecting the S domain and the GP fragment, as well as the C-terminal His-tag used for purification, are included, the total length of the displayed antigen approaches 500 amino acids. The ability of the S-GP_486_ fusion protein efficiently form PVNPs demonstrates the large cargo capacity of the norovirus SNP platform for antigen presentation. Structural modeling further suggested that the S-GP_486_ PVNP retain the characteristic architectural features of a typical PVNP, with multiple antigens displayed on its surface. These findings highlight the versatility of our SNP platform and suggest its broad applicability for the development of novel PVNPs displaying neutralizing antigens from diverse pathogens for vaccine development.

We observed clear differences in the antibody responses elicited by the PVNPs, as reflected by the detected IgG and IgA titers, when comparing ELISA results obtained using a baculovirus/insect cell-expressed GP protein as the capture antigen with those obtained using our bacterially expressed S-GP_486_ PVNP as the capture antigen. The higher antibody titers detected when using the S-GP_486_ PVNP as the capture antigen confirm the strong immune response induced by the PVNPs, likely attributable to the multivalent (60-, 180-, or 240-valent) display of repetitive antigens on the nanoparticle surface (Figure 4). In contrast, the lower antibody titers measured when using the baculovirus/insect cell-expressed trivalent GP protein (Figure 4) as the capture antigen may reflect differences in the post-translational modifications of GP proteins produced by the two expression systems (see below).

The native GP protein is known to be heavily glycosylated [29], and the baculovirus/insect cell expression system provides a favorable environment for the expressed GP protein to acquire at least partial glycosylation similar to its native form. By contrast, the *E. coli* expression system lacks the machinery required for protein glycosylation. Consequently, antibodies induced by the bacterially expressed S-GP PVNPs likely recognize the bacterially produced S-GP486 PVNP more efficiently because they share similar conformations due to the absence of glycosylation. Conversely, these antibodies may exhibit reduced recognition of the baculovirus/insect cell-expressed GP protein due to differences in glycosylation and, potentially, protein conformation. These findings suggest that future development of the S-GP PVNP vaccine candidates could benefit from the production in a eukaryotic expression system, such as mammalian, insect, or yeast cells, to better preserve native GP glycosylation patterns and potentially enhance the induction of antibodies that more closely recognize the native conformation of viral GP proteins.

In addition to glycosylation, disulfide bond formation is another important factor to consider when selecting an expression system for protein production. MARV GP contains both intra- and intermolecular disulfide bonds that are important for its structural integrity and biological function [8]. Although bacterial expression systems can support the formation of some disulfide bonds in recombinant proteins, they may not faithfully reproduce the complete native disulfide-bonding pattern found in eukaryotic cells. At this stage of development, we have not determined whether the bacterially expressed MARV GPs retain their native disulfide-bonded conformations. Therefore, a eukaryotic expression system may be a preferable option for future S-GP PVNP production, as it is more likely to reproduce native post-translational modifications, including proper disulfide bond formation and glycosylation (see above), thereby improving antigenicity and immunogenicity.

Similar to serum IgG, serum IgA is generally considered a marker of vaccine- or infection-induced immunity [30,31,32,33], and its protective role is likely distinct from that of serum IgG [34]. The role of MARV-specific serum IgA has not been as extensively characterized as it has for some respiratory or enteric viruses. We hypothesize that MARV GP-specific IgA can bind to the viral glycoprotein [35] and potentially interfere with virus–host cell interactions, thereby reducing infection. Therefore, we believe that evaluating GP-specific serum IgA, in addition to serum IgG, will provide a more comprehensive understanding of the immune responses elicited by the three S-GP PVNPs as potential vaccine candidates.

This study represents an early stage in the development of a novel MARV vaccine approach based on our proprietary PVNP platform and focused primarily on the generation and characterization of the S-GP PVNPs. As a result, we were unable to directly compare our vaccine candidates with the leading candidates currently being evaluated in the field. Nevertheless, our PVNP vaccine candidates may offer several potential advantages over the viral vector- and GP protein-based vaccine candidates currently prioritized by the WHO. First, the display of 60 to 240 copies of GP antigens on the surface of each PVNP (Figure 4) may enhance the immunogenicity of the PVNP-displayed GP proteins. Second, recombinant S-GP PVNPs can be produced using a scalable manufacturing process, potentially reducing vaccine production costs. Third, the nonreplicating nature of the PVNP vaccine eliminates the safety concerns associated with live viral vectors.

MARVs can be phylogenetically classified into two distinct lineages: the classical Lake Victoria MARV lineage, which includes the Angola, Musoke, Popp, and Ci67 strains, and the Ravn virus (RAVV) lineage. These lineages differ by approximately 22% at the overall protein sequence level [36,37,38]. MARV GPs are highly conserved among strains within the Lake Victoria MARV lineage, sharing over 90% amino acid identity [37], which supports the potential for cross-strain neutralization and protection [35]. The GP of the Angola strain has been widely used in MARV vaccine development because the Angola outbreak was the largest and most severe MARV outbreak reported to date [39], and the Angola isolate subsequently became the standard challenge strain in many preclinical efficacy studies [39]. Furthermore, the high degree of GP conservation within the Lake Victoria MARV lineage supports the potential for cross-reactive immune responses among classical MARV strains. However, cross-protection against the genetically distinct RAVV lineage remains to be experimentally evaluated.

The rationale for selecting the three GP fragments of different lengths (GP_278_, GP_377_, and GP_486_) was to explore the balance between maximizing the length of the GP antigen and maintaining the self-assembly capability of the resulting S-GP PVNPs, with the goal of incorporating the largest possible number of functional GP domains. Our results showed that all three PVNPs were successfully generated. As expected, the PVNP displaying the longest GP fragment (GP_486_) induced the strongest serum IgG and IgA antibody responses and was therefore identified as the lead vaccine candidate.

MARV is a Risk Group 4 (RG4) pathogen and is classified as a Biosafety Level 4 (BSL-4) agent, requiring the highest level of biological containment. Therefore, our laboratory is not equipped to evaluate the protective efficacy of the S-GP PVNP vaccine candidates against live MARV challenge, which represents a limitation of this study. Future studies will involve collaborations with experts who have established MARV pseudovirus neutralization assays and with investigators capable of conducting MARV challenge studies in appropriate animal models to assess the neutralizing activity and protective efficacy of our S-GP PVNP vaccine candidates. In addition, we were unable to perform cytokine profiling to assess the potential immunological impact of the nanoparticle treatment. This represents an additional limitation of the current study and will be addressed in future investigations. On the other hand, although this was not the primary focus of our work, a recent study demonstrated that antibodies induced by SNP were capable of neutralizing human norovirus replication in a human intestinal enteroid system, indicating that norovirus S domain is a neutralizing antigen [40]. In the present study, we observed that the S-GP PVNPs also induced SNP-specific IgG and IgA responses. These findings raise the possibility that the S-GP PVNPs could function as a dual vaccine against both MARV and norovirus, a hypothesis that merits further investigation.

## Data Availability

The vast majority of the relevant data has been published in this manuscript. Any additional data are available upon request.

## Abbreviations

The following abbreviations are used in this manuscript:

MARV	Marburg virus
EBOV	Ebolavirus
MVD	Marburg virus disease
NP	Nucleoprotein
PVNP	Pseudovirus nanoparticle
VP	Viral protein
GP	Glycoprotein
S	Shell domain
P	Protruding domain
SNP	Shell nanoparticle
S-GP	S-domain-glycoprotein fusion protein
IPTG	Isopropyl ß-D-1-thiogalactoyranoside
GnHCl	Guanidine hydrochloride
SDS-PAGE	Sodium dodecyl sulfate polyacrylamide gel electrophoresis
FPLC	Fast-performance liquid chromatography
GST	Glutathione S-transferase
TEM	Transmission electron microscopy
DLS	Dynamic light scattering
cryoEM	Cryogenic electron-microscopy
HRP	Horseradish Peroxidase
OD	Optical density
CCHMC	Cincinnati Children’s Hospital Medical Center
ELISA	Enzyme-linked immunosorbent assays
IACUC	Institutional Animal Care and Use Committee
NIH	National Institute of Health
NIAID	National Institute of Allergy and Infectious Diseases
kDa	Kilodalton
HA	Hemagglutinin
UCSF	University of California, San Francisco
